# Challenges of Diagnosing Acute HIV-1 Subtype C Infection in African Women: Performance of a Clinical Algorithm and the Need for Point-of-Care Nucleic-Acid Based Testing

**DOI:** 10.1371/journal.pone.0062928

**Published:** 2013-04-30

**Authors:** Koleka Mlisana, Magdalena Sobieszczyk, Lise Werner, Addi Feinstein, Francois van Loggerenberg, Nivashnee Naicker, Carolyn Williamson, Nigel Garrett

**Affiliations:** 1 Centre for the AIDS Programme of Research in South Africa (CAPRISA), University of KwaZulu-Natal, Durban, South Africa; 2 Department of Medical Microbiology, University of KwaZulu-Natal, Durban, South Africa; 3 National Health Laboratory Services, Durban, South Africa; 4 Columbia University, New York, New York, United States of America; 5 Institute of Infectious Diseases and Molecular Medicine & the Division of Medical Virology, University of Cape Town, Cape Town, South Africa; University of Toronto, Canada

## Abstract

**Background:**

Prompt diagnosis of acute HIV infection (AHI) benefits the individual and provides opportunities for public health intervention. The aim of this study was to describe most common signs and symptoms of AHI, correlate these with early disease progression and develop a clinical algorithm to identify acute HIV cases in resource limited setting.

**Methods:**

245 South African women at high-risk of HIV-1 were assessed for AHI and received monthly HIV-1 antibody and RNA testing. Signs and symptoms at first HIV-positive visit were compared to HIV-negative visits. Logistic regression identified clinical predictors of AHI. A model-based score was assigned to each predictor to create a risk score for every woman.

**Results:**

Twenty-eight women seroconverted after a total of 390 person-years of follow-up with an HIV incidence of 7.2/100 person-years (95%CI 4.5–9.8). Fifty-seven percent reported ≥1 sign or symptom at the AHI visit. Factors predictive of AHI included age <25 years (OR = 3.2; 1.4–7.1), rash (OR = 6.1; 2.4–15.4), sore throat (OR = 2.7; 1.0–7.6), weight loss (OR = 4.4; 1.5–13.4), genital ulcers (OR = 8.0; 1.6–39.5) and vaginal discharge (OR = 5.4; 1.6–18.4). A risk score of 2 correctly predicted AHI in 50.0% of cases. The number of signs and symptoms correlated with higher HIV-1 RNA at diagnosis (r = 0.63; p<0.001).

**Conclusions:**

Accurate recognition of signs and symptoms of AHI is critical for early diagnosis of HIV infection. Our algorithm may assist in risk-stratifying individuals for AHI, especially in resource-limited settings where there is no routine testing for AHI. Independent validation of the algorithm on another cohort is needed to assess its utility further. Point-of-care antigen or viral load technology is required, however, to detect asymptomatic, antibody negative cases enabling early interventions and prevention of transmission.

## Introduction

The devastating toll of the HIV/AIDS epidemic on sub-Saharan Africa, with 67% of the estimated 33.3 million world-wide HIV infections in this region alone, is well recognized [1]. Individuals with acute HIV infection (AHI) have been shown to be more infectious compared to individuals with chronic infection. Data from several cohorts provide estimates of HIV transmission per coital act in sub-Saharan Africa, with dramatically higher transmission rates during acute and early, compared to latent or chronic infection [2]–[4]. One study modeling the impact of antiretroviral therapy (ART) on the HIV epidemic estimated that 38.4% of ongoing HIV transmissions are attributable to sexual contact with early HIV index cases [5]. This is presumably due to several factors including very high plasma and genital tract HIV viral load [6]–[9]. Early identification of individuals in the acute/early stage of infection would not only enable prompt intervention to prevent transmission to negative partners, but may also allow for earlier initiation of treatment. Data on the clinical benefits of ART in patients with acute HIV are conflicting [10]–[12]. Theoretical benefits of this intervention include potentially preserving immune function, reducing the pool of latently infected CD4 cells, thereby altering the virologic set point and delaying disease progression. These have to be weighed against side effects and patient readiness to start antiretrovirals [3], [13]–[17].

Making the diagnosis of acute HIV infection is challenging since the signs and symptoms of acute retroviral syndrome are non-specific. Of note, data on clinical characteristics of subtype C infection, particularly among women, are currently limited [18], [19]. Days to weeks after acquisition, 40–90% of individuals develop signs and symptoms including a flu-like illness or mononucleosis-like syndrome [20]. The onset of symptoms coincides with viral replication and peak viremia [21]–[23]. During this window period, only HIV RNA can be detected in plasma until the appearance of p24 antigen and, subsequently, HIV antibodies. Laboratory-based fourth generation Ag/Ab combination assays improve detection of acutely-infected individuals compared to third generation antibody assays. However, in resource limited, high prevalence settings, health services rely predominantly on point-of-care testing (POCT) and, disappointingly, a recent evaluation found that the antigen component of the fourth generation Determine® HIV-1/2 Ag/Ab Combo rapid test performed poorly in detecting acute infection [24]. Initiating RNA testing still requires clinician’s high index of suspicion and availability of resources. Thus, a clinical algorithm which prioritizes nucleic acid HIV testing would be of benefit, especially in decentralized health care settings.

The study, conducted by the Centre for the AIDS Programme of Research in South Africa (CAPRISA), was a prospective cohort study to examine the pathogenesis of acute HIV subtype C infection and to describe the immunologic, virologic and clinical characteristics of acute and early infection [25]. The purpose of the current analysis was (i) to describe the signs and symptoms of AHI in order to develop a diagnostic model for identifying cases with high probability of AHI which would allow interventions like targeted HIV-1 RNA or p24 antigen testing; (ii) to calculate sensitivity and specificity of the risk score to predict presence or absence of AHI; (iii) to correlate clinical signs and symptoms of AHI with early virologic and clinical progression of HIV.

## Methods

### Ethics Statement

Ethics approval for the study was obtained from the University of KwaZulu-Natal and the University of Cape Town. Written informed consent was obtained from all participants.

### Cohort

Between August 2004 and May 2005, a cohort of 245 HIV-uninfected women at high risk of infection was recruited in Durban, South Africa [25]. Women were considered high-risk if they reported having more than three sexual partners in the past 3 months or self-identified as sex workers.

### Study Evaluations and Definition of Acute HIV Infection

Demographic and behavioural questionnaires were administered at enrollment. Women were evaluated at baseline and monthly thereafter with standardized interviews using a locally developed Clinical Evaluation Tool (CET) to screen for signs and symptoms associated with AHI. Interviews were performed by the research clinician who either spoke the local language or was assisted by a nurse who acted as an interpreter. Signs and symptoms screened for included: fever, fatigue, lethargy, night sweats, rash, headache, swollen lymph glands, sore throat, myalgia, any swollen joints, morning stiffness (joints), nausea, vomiting, diarrhoea, mucocutaneous ulceration, gingivitis, loss of appetite, reported weight loss, confusion, photophobia, neck stiffness, retro-orbital pain and vaginal discharge. Women also underwent a physical examination to determine the presence or absence of fever (>38°C), measured weight loss, evidence of rash, lymphadenopathy, pharyngitis, thrush, mucocutaneous ulceration, conjunctivitis, hepatomegaly and splenomegaly. Whilst sexually transmitted infections (STIs) were managed syndromically, six monthly laboratory screening for STIs was performed as well.

The CET was administered before the HIV status was established. HIV testing algorithm consisted of two rapid HIV-1 antibody tests: the Determine HIV-1 test (Abbott Diagnostics, Johannesburg, South Africa) and the Capillus HIV-1/HIV-2 test (Trinity Biotech, USA). Discordant rapid antibody results were confirmed by HIV enzyme immunoassay (EIA) (BEP 2000; Dade Behring, Marburg, Germany). A pooled plasma HIV-1 RNA (AMPLISCREEN™ HIV-1 Tests, v1.5– Roche Diagnostics) was performed at enrollment and each visit subsequently, irrespective of antibody result. A primary pool containing 24 samples was used and if positive disaggregated into smaller secondary pools. Diagnosis of AHI was made if a woman had detectable plasma HIV RNA level (using <400 copies/mL lower limit of detection assay) and/or a positive HIV antibody test following a previously documented negative test. Women presenting for unscheduled visits also completed a CET and were tested for HIV following the same algorithm. Participants underwent risk reduction counseling and were provided with male and female condoms at every visit.

### Data Analysis

Signs and symptoms reported on the CET at all HIV-negative visits and the AHI visit were used in the analysis. Prevalence of signs and symptoms at both the AHI and non-AHI visits was assessed. Weight before and after seroconversion was compared using a Wilcoxon signed rank test. A Pearson’s correlation coefficient was calculated to assess the strength of the linear relationship between the number of signs and symptoms and viral load, as well as CD4 count, measured at seroconversion, 6 and 12 months post infection. Signs/symptoms at the AHI visit were compared to those captured on the CET at HIV-negative visits from both seroconverters and those who remained HIV negative. To determine which signs/symptoms were associated with AHI, symptoms in women who seroconverted were compared with symptoms in women who remained HIV negative. Odds ratios (OR) for each of the signs/symptoms were calculated using a generalized estimating equation model, using a binomial distribution, accounting for multiple visits for the same participant. Two adjusted models were fitted to assess clinical factors predictive of AHI. Variables which were associated with AHI at p<0.15 in each of the models were then included in the final model, which was used to develop a model-based score to predict AHI. In addition to applying a risk score model to predict AHI, the absolute number of signs/symptoms was also assessed to determine association with AHI. Sensitivity, specificity and positive likelihood ratios (with 95% confidence intervals) for detecting AHI were calculated for different numbers of signs and symptoms. This was also repeated excluding POCT negative, RNA positive participants reflecting current clinical practice in South Africa where RNA testing is not part of the algorithm for HIV diagnosis. All data analysis was performed using SAS version 9.2 (SAS Institute Inc., Cary).

## Results

### Demographic Characteristics of the Cohort

Between August 2004 and May 2005, 775 high risk women were screened and 245 HIV uninfected women were enrolled and followed monthly for two years. The demographic and behavioural characteristics of the 245 women have been described in detail elsewhere [25] and are summarized in Table 1. Briefly, the median age of the participants was 36 (interquartile range (IQR): 24–42) years, 41.2% had at least 11 years of education, 58.8% reported using a condom at last sexual encounter, 13.9% and 35.0% indicated that they were never able to insist on condom use with casual and steady partners, respectively [26]. Most women (85.4%) reported having 2–5 casual partners in the 3 months prior to enrollment and 78.8% were self-identified commercial sex workers. Of note, the prevalence of HIV among women screening for enrollment into the HIV negative cohort was 59.6% [25].

**Table 1:** Baseline demographic and behavioural characteristics of South African women at high risk of HIV acquisition stratified by HIV status [25].

Characteristic	All % (N) (N = 245)	HIV negative % (N)(N = 217)	AHI# cases % (N) (N = 28)	
**Age in years**				
≥25	73.5% (180)	75.6% (164)	57.1% (16)	
<25	26.5% (65)	24.4% (53)	42.9% (12)	
**Relationship status**				
No partner (single, widowed, divorced)	7.4% (18/244)	7.9% (17/216)	3.6% (1)	
One partner (stable or married)	35.7% (87/244)	37.5% (81/216)	21.4% (6)	
Many partners	57.0% (139/244)	54.6% (118/216)	75.0% (21)	
**Number of casual partners in the last 3 months (baseline)**				
0–1	5.0% (12/240)	4.7% (10/212)	7.1% (2)	
2–5	85.4% (205/240)	85.9% (182/212)	82.1% (23)	
>5	9.6% (23/240)	9.4% (20/212)	10.7% (3)	
Proportion of commercial sex workers	78.8% (193)	79.7% (173)	71.4% (20)	
**Highest level of school completed**				
Grade <8	24.5% (60)	26.3% (57)	10.7% (3)	
Grade 8–10	34.3% (84)	32.3% (70)	50.0% (14)	
Grade 11–12	41.2% (101)	41.5% (90)	39.3% (11)	
Mean years sexually active (SD)	17.2 (10.38)	17.7 (10.35)	13.5 (10.08)	
**Age at sexual debut**				
<16	28.2% (69)	26.7% (58)	39.3% (11)	
≥16	71.8% (176)	73.3% (159)	60.7% (17)	
**Partner type at last sexual act (baseline)**				
Steady Partner	75.9% (186)	77.9% (169)	60.7% (17)	
Casual Partner	24.1% (59)	22.1% (48)	39.3% (11)	
Proportion with condom use at last sex act	58.8% (144)	58.1% (126)	64.3% (18)	

# Acute HIV Infection.

### Signs and Symptoms in Acute HIV Infection

The 245 HIV negative women had 4845 HIV negative visits, scheduled monthly, with a missed visit rate of 3.9%. The median time between visits was 28 days (IQR 28 - 34 days). Twenty-eight women seroconverted after a total of 390 person-years of follow-up with an HIV incidence of 7.2/100 person-years (95%CI: 4.5–9.8).

The frequency of signs/symptoms reported at AHI visit was higher than that reported at the HIV-negative visits (Table 2). The most prevalent signs/symptoms reported at AHI visit were loss of appetite (28.6% vs. 2.1% in HIV uninfected, p<0.001), headache (25.0% vs. 5.7%, p<0.001), rash (25.0% vs. 2.9%, p<0.001), sore throat (21.4% vs. 2.1%, p<0.001), fever (17.9% vs. 2.9%, p<0.001), arthralgia (17.9% vs. 2.9%, p<0.001), and reported weight loss (21.4% vs. 1.3%, p<0.001). Of note, of the 28 acute infections, 60.7% (n = 17) had confirmed (measured) weight loss in the preceding two weeks, with a median reduction of 2.0 kg (IQR 1.3–2.6 kg). The median weight loss during seroconversion for all women with acute infection was 1.3 kg (IQR 0.0–2.2 kg; p = 0.025).

**Table 2:** Signs and symptoms associated with acute HIV infection.

Signs or symptoms	Total cohort HIV negative visitsN (%) (N = 4845)		AHI cases N (%)(N = 28)		*Unadjusted Odds Ratio(95% CI)	p-value	Sensitivity (%)	Specificity (%)
Headache	277	(5.7)	7	(25.0)	5.5 (2.3–13.0)	<0.001	25.0	94.3
Fever	140	(2.9)	5	(17.9)	7.3 (2.7–19.5)	<0.001	17.9	97.1
Sore throat	100	(2.1)	6	(21.4)	12.9 (5.1–32.6)	<0.001	21.4	97.9
Night sweats	119	(2.5)	3	(10.7)	4.8 (1.4–16.0)	0.012	10.7	97.5
Fatigue	133	(2.7)	7	(25.0)	11.8 (4.9–28.3)	<0.001	25.0	97.3
Swollen glands	280	(5.8)	3	(10.7)	2.0 (0.6–6.5)	0.275	10.7	94.2
Rash	138	(2.8)	7	(25.0)	11.4 (4.8–27.2)	<0.001	25.0	97.2
Loss of appetite	103	(2.1)	8	(28.6)	18.4 (7.9–42.8)	<0.001	28.6	97.9
Weight loss	63	(1.3)	6	(21.4)	20.7 (8.1–52.8)	<0.001	21.4	98.7
Nausea	80	(1.7)	5	(17.9)	12.9 (4.8–34.9)	<0.001	17.9	98.3
Diarrhoea	102	(2.1)	4	(14.3)	7.8 (2.6–22.7)	<0.001	14.3	97.9
Arthralgia	139	(2.9)	5	(17.9)	7.4 (2.8–19.6)	<0.001	17.9	97.1
Myalgia	47	(1.0)	3	(10.7)	12.3 (3.6–42.0)	<0.001	10.7	99.0
Lethargy	49	(1.0)	3	(10.7)	11.7 (3.4–40.2)	<0.001	10.7	99.0
Oral ulcers	48	(1.0)	2	(7.1)	7.7 (1.8–33.3)	0.006	7.1	99.0
Genital ulcers	8	(0.2)	2	(7.1)	46.5 (9.4–229.6)	<0.001	7.1	99.8
Vaginal discharge	91	(1.9)	3	(10.7)	6.3 (1.9–21.1)	0.003	10.7	98.1

* Odds Ratio adjusted for repeated measures.

Note: Signs and symptoms reported with <5% frequency in the AHI cases or p>0.15 were omitted. These included splenomegaly, anal ulcers, swollen joints, gingivitis, observed fever, aseptic meningitis, peripheral neuropathy, conjunctivitis, hepatomegaly, photophobia, parasthaesia, retro-orbital headache and neck stiffness.

Fifty-seven percent of participants with AHI reported at least one sign or symptom and the specificity and likelihood ratios increased with an increasing number of reported symptoms (Table 3). Participants with one to three signs/symptoms were more likely to have AHI compared to women with no signs/symptoms (OR = 3.9; 95% CI: 1.6–9.2; p = 0.002); and presence of ≥4 signs/symptoms was strongly associated with AHI (OR = 17.3; 95% CI: 6.7–44.7; p<0.001). When excluding all POCT Ab negative, RNA positive AHI cases, the prevalence of signs/symptoms increased to 76.5% and the association with AHI was even stronger (1–3 symptoms: OR = 10.3; 95% CI: 3.1–34.5, ≥4 symptoms: OR = 36.9. 95% CI: 9.8–140). When comparing AHI and non-AHI visits among women who seroconverted, loss of appetite, fatigue, headache, rash and sore throat remained the most commonly reported symptoms.

**Table 3:** Sensitivity and Specificity for different number of signs and symptoms.

Number of symptoms	Sensitivity (%)	Specificity (%)	+LR (95% CI)
**All AHI cases included (N = 28)**			
≥1	57.1%	81.5%	3.09 (2.23–4.28)
≥2	42.9%	91.4%	4.98 (3.22–7.71)
≥3	32.1%	95.3%	6.86 (3.95–11.93)
≥4	25.0%	97.3%	9.11 (4.69–17.68)
≥5	25.0%	98.1%	13.31 (6.79–26.09)
**Excluding POCT** * **negative, RNA positive cases (N = 17)**			
≥1	76.5%	81.5%	4.13 (3.15–5.42)
≥2	58.8%	91.4%	6.82 (4.54–10.27)
≥3	41.2%	95.3%	8.76 (4.89–15.69)
≥4	29.4%	97.2%	10.65 (5.00–22.68)
≥5	29.4%	98.1%	15.68 (7.30–33.69)

* POCT: point of care test.

### Factors Predictive of Acute HIV Infection

In an adjusted model, six predictors of AHI were identified: age <25 years (OR = 3.2, 95% CI: 1.4–7.1), rash (OR 6.1, 95% CI: 2.4–15.4), sore throat (OR 2.7, 95% CI: 1.0–7.6), loss of appetite (OR 4.7, 95% CI: 1.4–15.6), weight loss (OR 4.4, 95% CI: 1.5–13.4), vaginal discharge (OR: 5.4, 95% CI: 1.6–18.4) and genital ulcer (OR 8.0, 95% CI: 1.6–39.5) (Table 4). In this cohort, behavioural factors, including number of partners, condom use, whether the partner was casual or steady, and educational level were not associated with AHI in the final model (data not shown).

**Table 4:** Unadjusted and adjusted analysis of signs and symptoms associated with acute HIV Infection.

Predictor	Unadjusted Odds Ratio(95% CI)	Adjusted* Odds Ratio(95% CI)	Risk score model**: Adjusted Odds Ratio (95%CI)
**Demographics**			
Age			
≥25	1.0	1.0	1.0
<25	2.77 (1.30–5.86)	2.60 (1.23–5.50)	3.15 (1.41–7.07)
**Symptoms and Signs**			
Headache	5.50 (2.32–13.04)	–	–
Fever	7.31 (2.74–19.50)	–	–
Sore Throat	12.94 (5.14–32.61)	3.16 (1.01–9.83)	2.73 (0.99–7.56)
Night Sweats	4.77 (1.42–16.00)	–	–
Fatigue	11.81 (4.93–28.26)	–	–
Swollen Glands	1.96 (0.59–6.52)	–	–
Rash	11.37 (4.75–27.19)	5.42 (2.10–13.99)	6.07 (2.39–15.43)
Loss of Appetite	18.42 (7.93–42.78)	4.91 (1.40–17.29)	4.72 (1.42–15.63)
Weight Loss	20.70 (8.12–52.80)	3.50 (1.17–10.41)	4.43 (1.46–13.43)
Nausea	12.95 (4.80–34.91)	–	–
Diarrhoea	7.75 (2.64–22.74)	–	–
Athralgia	7.36 (2.76–19.64)	–	–
Myalgia	12.25 (3.58–41.98)	–	–
Lethargy	11.74 (3.43–40.19)	–	–
Oral Ulcers	7.69 (1.77–33.30)	–	–
Genital Ulcers	46.51 (9.42–229.60)	8.11 (1.97–33.49)	7.95 (1.60–39.49)
Vaginal Discharge	6.27 (1.86–21.13)	6.19 (1.73–22.15)	5.38 (1.57–18.40)

* Model for behavioural and demographic factors adjusted for age in years, relationship status, education and age at sexual debut (data not shown), while model for symptoms and signs adjusted for sore throat, rash, loss of appetite, weight loss, genital ulcers and vaginal discharge.

** Risk score model adjusting for age, sore throat, rash, loss of appetite, weight loss, genital ulcers and vaginal discharge.

### Signs and Symptoms According to HIV Diagnostic Test

All participants had two POCTs and a viral load RNA performed at each visit. Positive results were followed up with a laboratory-based third generation ELISA test. Of the 28 women with AHI, 17 (60.7%) had positive POCTs including one participant with discordant POCTs, while all were HIV positive on RNA testing (Table 5). Three-quarters of women (13/17; 76.5%) with positive or discordant POCTs were symptomatic compared to only one quarter with two negative POCTs at AHI (3/11; 27.3%, p = 0.019) who would have been identified with our clinical algorithm. However, it is important to note that, 8/28 women (28.6%) were POCT negative and experienced no signs or symptoms at the AHI visit and would therefore not have been identified by POCT testing or any clinical evaluation of AHI symptoms.

**Table 5:** Signs and symptoms according to HIV diagnostic test.

HIV diagnostic testing	N (%) of AHI cases (N = 28)	N (%) with no symptoms	N (%) with any symptoms	p-value*
POCT antibody positive or discordant	17 (60.7%)	4/17 (23.5%)	13/17 (76.5%)	0.0189
POCT antibody negative	11 (39.3%)	8/11 (72.7%)	3/11 (27.3%)	–

* Fisher’s Exact test.

### Risk-score Model Predicting Acute HIV Infection

The variables significantly associated with AHI in the adjusted analysis were used to construct a predictor risk score for AHI using the regression coefficients from the final adjusted model. The signs/symptoms in the model were weighted by the regression coefficients, rounded off to the nearest integer. Hence, the risk score algorithm was calculated as follows:
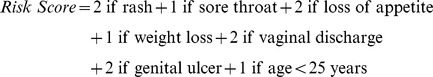



A risk score was calculated for each visit. The sensitivity and specificity of different cut-off scores were determined by comparing the risk score with the HIV status (HIV-1 RNA and/or antibody positive) of the participant at every visit. Simply RNA PCR testing every person with at least one symptom would yield a sensitivity of 57.1%. A risk score of at least 1 occurred at 28.0% of participant visits. Having a score of at least 1, that is, presenting with any of the risk score symptoms or being under 25 years of age, would identify 67.9% cases of AHI and positive predictive value (PPV) of the test would be low at 1.4%. Using this scoring system, a score of 2 could identify 50.0% of AHI cases by testing only 7.3% of participants, with a PPV of 3.9%. At a cut-off of 3, only 3.0% of participants would be tested to identify 39.3% of all AHI, with a PPV of 7.5%.

### Correlation of Number of Signs and Symptoms and Markers of HIV Disease Progression

There was a significant positive correlation between the number of signs/symptoms and the HIV-1 plasma viral load at the AHI visit (correlation coefficient of 0.63, p = <0.001) with participants who had a high viral load at seroconversion having a greater number of signs/symptoms. However, this trend did not persist, and no correlation was observed between the initial number of signs/symptoms and HIV-1 plasma viral loads at 6 or 12 months. There was no statistically significant correlation between the number of signs/symptoms and CD4 count at seroconversion (correlation coefficient of 0.16, p = 0.41). There was also no significant difference in the duration of signs/symptoms and self-reported severity of diarrhoea, skin rash, lethargy and pharyngitis (mild to severe) between women with AHI and HIV-negative women (data not shown).

## Discussion

The period of acute HIV infection contributes to a considerable proportion of transmission events and, therefore, identifying those who are acutely infected is of paramount importance in curbing the epidemic [20]. Accurate diagnosis of acute HIV infection includes use of molecular techniques like HIV RNA PCR detection which may either be expensive or not readily available in some settings.

In this prospective cohort study of 245 high-risk women we identify clinical signs and symptoms predictive of acute subtype C infection. We present a screening algorithm which can be used to enhance detection of acute HIV infection in a resource-limited setting. Importantly, we report that younger age, rash, sore throat, loss of appetite, weight loss and vaginal discharge or genital ulcers were associated with AHI. A similar constellation of symptoms has been reported in other studies [27]–[32], although in our cohort none of the signs or symptoms had prevalence above 28.6%. In fact, fever, commonly reported in other studies, was found in only 17.9% of our participants [27]–[32]. Most available data, however, pertains to predominantly male cohorts with subtype B infection.

Data in the sub-Saharan African context is more limited [18], [19], [33], particularly among women with subtype C infection. Available literature on acute infection reports higher prevalence of symptoms than what was noted in our cohort. For example, in a prospective cohort of female sex workers in Kenya, 81% of women had at least one symptom, in comparison to only 57.1% in our cohort [18]. More recently, in a cross-sectional cohort of predominantly male adults presenting with fever in Mozambique, among those identified with acute infection, all had reported sore throat and 67% complained of a mononucleosis-like syndrome. The limitation of this particular study, however, was a small number of individuals with acute HIV infection and that the administration of the questionnaire a week after the diagnosis visit may have influenced the findings [34].

Our AHI cohort included individuals who were diagnosed by RNA testing alone potentially before a possible seroconversion illness could have manifested itself. Excluding these cases, the number of women who reported signs/symptoms was 76.5% similar to what has been previously reported [18], [19]. Other contributing reasons for the observed difference may have been cultural or gender-specific characteristics in reporting signs/symptoms, or differences in how HIV subtypes manifest disease [35]. In addition, the fact that our participants were not being evaluated in an STI clinic or referred for a particular complaint, meant that they were probably less likely to report signs/symptoms. It is therefore possible that if the clinical algorithm was applied to a primary health care facility or STI clinic, the sensitivity and specificity of the algorithm would improve, as well as its utility as part of an HIV screening initiative in these settings.

This study demonstrates that it is possible to create a model predictive of AHI in this population. RNA-PCR testing everyone with a risk cut-off of ≥1 (28.0% of participant visits), would allow for the identification of 67.9% of acutely-infected individuals. In contexts of limited resources, the cut-off score could be increased, thus reducing the number of tests done in order to identify acute infection. In our cohort, testing only women with a risk score of ≥2 would result in RNA-PCR testing of only 7.3% of participant visits, and half (50.0%) of all acute infections would be identified. The costs of these additional tests could be offset by the benefit of being able to intervene early in acutely-infected individuals, to initiate care and help prevent HIV transmission during this high-risk period.

Our algorithm did not yield as high a predictive value or sensitivity as the algorithm created from a cross-sectional assessment of individuals presenting at an STI clinic in Malawi. Powers et al, who included discordant POCT results in their model, were able to identify 95% of AHI cases by performing RNA or p24 testing in 40% of their population [19]. Apart from a difference in testing methodologies between the two studies, it is possible that the models were affected by differences in the population with higher overall prevalence of signs/symptoms such as genital ulcer disease in individuals presenting for diagnosis and treatment at an STI clinic in the Malawi study.

Many demographic factors such as number of sexual partners, age at sexual debut, education level and condom use were not significantly associated with AHI in our cohort. A possible explanation is that behavioural risk assessment was performed only at enrollment and thus, because of ongoing risk reduction counseling, it is possible that women modified their risk behaviour over the course of the study resulting in a decrease of HIV incidence over time [25]. Only age <25 years was strongly predictive of acute HIV infection (OR 3.2). This finding reflects what has been reported in other studies that indicate a higher incidence of HIV in younger women [36].

Availability of longitudinal follow-up data from the immediate post-seroconversion period, allowed us to demonstrate a correlation between the number of signs/symptoms and early HIV RNA levels. While in our study this association did not persist beyond the early HIV infection period, previous studies found an association between the number, severity and duration of symptoms and higher viral load set-points in patients infected with subtype B virus [37]–[39]. For example, one cohort study with over 600 individuals found that HIV RNA levels remained persistently higher in symptomatic compared to asymptomatic patients highlighting the need to identify those individuals and ensure access to care [40]. Data from sub-Saharan Africa is limited but our findings differ from what has been reported in a cohort of women in Kenya where higher set-point viral load and more severe illness at acute HIV infection predicted faster disease progression [41], [42].

Our study demonstrates that a considerable proportion of individuals (28.6%) neither test positive on conventional point-of-care testing nor report any symptoms when presenting to testing facilities with acute HIV infection. This population provides a true challenge for the scientific community to develop reliable fourth generation Ag/Ab combination POCTs or low-cost point-of-care viral load technology. Although the Determine® HIV-1/2 Ag/Ab Combo rapid test performed poorly in detecting acute infection in a recent trial [24] we hope that this is not the end of POCT Ag technology. Furthermore, several POCT viral load assays are currently being evaluated including the SAMBA (simple amplification based assay; Diagnostics Development Unit University of Cambridge) a semi quantitative test for using isothermal amplification and visual detection by dipstick, the Liat TM Analyser, manufactured by IQuum (Marlborough, MA), is a real-time, battery operated, small, portable PCR kit providing quantitative results and the Alere NAT system (Alere Technologies, Stirling, UK). The hope would be that the implementation of this technology, primarily developed in order to monitor HIV positive patients on ART, will also allow health care providers in less developed settings to test for AHI.

The strengths of our study rest in the fact that this is a longitudinal cohort composed of women with subtype C infection which was conducted in a non-STI clinic setting and therefore may be more representative of the general population. The cohort design included clinical assessment at every visit which allowed comparison of signs/symptoms pre-seroconversion to AHI visit. Given the poor specificity of signs/symptoms and concurrent background of other illnesses, this systematic clinical assessment is an advantage of this study. In contrast to previous studies, all participants underwent antibody and RNA PCR testing at every visit allowing a detailed analysis of the characteristics of these tests and correlation with symptoms.

Our study highlights the challenges associated with recognizing and diagnosing acute HIV infection in a cohort of women at high risk of infection. Although signs/symptoms of AHI are non-specific and highly variable, it is possible to identify a large proportion of individuals who have acute or early HIV infection if there is a high index of suspicion to consider the diagnosis and initiate appropriate testing. More advanced laboratory diagnostic tools offer promise of earlier detection of incident infections [21], but even then clinical judgement guiding prioritization of testing will remain critical, especially in resource-limited settings. Our risk score model based on several signs/symptoms most predictive of AHI may allow identification of women likely to be acutely infected with HIV and prioritize testing with RNA or newer generation assays, ultimately reducing cost of AHI diagnosis. Further independent validation of the model in different settings and larger sero-incidence cohorts would be important to assess the utility of the model in practice. In a setting where nucleic acid testing is not yet readily available, the ability to develop a checklist of signs/symptoms that would increase clinical suspicion of AHI would be of benefit for triaging of high-risk women and may lead to earlier detection of infection.

A recent study by Powers and colleagues concluded that in order to have a large impact on decreasing HIV prevalence, it is critical to identify and engage in care individuals in acute and early stages of HIV infection [5]. Thus, rapid detection of incident infections is an important component of test-and-treat and other prevention strategies. Screening for AHI is, however, associated with substantial costs of using newer generation HIV diagnostics and adds operational challenges [43]. Until rapid and cheap point-of-care assays which reliably detect HIV RNA or p24 antigen are commercially available and deployable in resource-limited settings, detecting AHI will remain a challenge. In the meantime, a clinically relevant risk algorithm which would decrease the number of individuals needed to screen with laboratory tests to detect AHI, is critical. The window of opportunity to intervene is narrow but the potential to benefit the individual and prevent onward transmission is considerable.

## References

[1] UNAIDS (2010) Report on the global HIV/AIDS epidemic 2010. UNAIDS.

[2] Boily MC, Baggaley RF, Wang L, Masse B, White RG, et al (2009) Heterosexual risk of HIV-1 infection per sexual act: systematic review and meta-analysis of observational studies. Lancet Infect Dis 9: 118–129. 19179227 10.1016/S1473-3099(09)70021-0 PMC4467783

[3] Cohen MS, Shaw GM, McMichael AJ, Haynes BF (2011) Acute HIV-1 Infection. N Engl J Med 364: 1943–1954. 21591946 10.1056/NEJMra1011874 PMC3771113

[4] Wawer MJ, Gray RH, Sewankambo NK, Serwadda D, Li X, et al (2005) Rates of HIV-1 transmission per coital act, by stage of HIV-1 infection, in Rakai, Uganda. J Infect Dis 191: 1403–1409. 15809897 10.1086/429411

[5] Powers KA, Ghani AC, Miller WC, Hoffman IF, Pettifor AE, et al (2011) The role of acute and early HIV infection in the spread of HIV and implications for transmission prevention strategies in Lilongwe, Malawi: a modelling study. Lancet 378: 256–268. 21684591 10.1016/S0140-6736(11)60842-8 PMC3274419

[6] Gray RH, Wawer MJ, Brookmeyer R, Sewankambo NK, Serwadda D, et al (2001) Probability of HIV-1 transmission per coital act in monogamous, heterosexual, HIV-1-discordant couples in Rakai, Uganda. Lancet 357: 1149–1153. 11323041 10.1016/S0140-6736(00)04331-2

[7] Pilcher CD, Joaki G, Hoffman IF, Martinson FE, Mapanje C, et al (2007) Amplified transmission of HIV-1: comparison of HIV-1 concentrations in semen and blood during acute and chronic infection. AIDS 21: 1723–1730. 17690570 10.1097/QAD.0b013e3281532c82 PMC2673564

[8] Quinn TC, Wawer MJ, Sewankambo N, Serwadda D, Li C, et al (2000) Viral load and heterosexual transmission of human immunodeficiency virus type 1. Rakai Project Study Group. N Engl J Med 342: 921–929. 10738050 10.1056/NEJM200003303421303

[9] Hughes JP, Baeten JM, Lingappa JR, Magaret AS, Wald A, et al (2012) Determinants of per-coital-act HIV-1 infectivity among African HIV-1-serodiscordant couples. J Infect Dis 205: 358–365. 22241800 10.1093/infdis/jir747 PMC3256946

[10] Fidler S (2011) The effect of short-course antiretroviral therapy in primary HIV infection: final results from an international randomised controlled trial; SPARTAC. 6th International AIDS Society Conference on HIV pathogenesis, treatment and prevention. Rome, Italy.

[11] Grijsen ML, Steingrover R, Wit FW, Jurriaans S, Verbon A, et al (2012) No treatment versus 24 or 60 weeks of antiretroviral treatment during primary HIV infection: the randomized Primo-SHM trial. PLoS Med 9: e1001196. 22479156 10.1371/journal.pmed.1001196 PMC3313945

[12] Hogan CM, Degruttola V, Sun X, Fiscus SA, Del Rio C, et al (2012) The setpoint study (ACTG A5217): effect of immediate versus deferred antiretroviral therapy on virologic set point in recently HIV-1-infected individuals. J Infect Dis 205: 87–96. 22180621 10.1093/infdis/jir699 PMC3242744

[13] Bell SK, Little SJ, Rosenberg ES (2010) Clinical management of acute HIV infection: best practice remains unknown. J Infect Dis 202 Suppl 2S278–288. 20846034 10.1086/655655 PMC6037306

[14] Chun TW, Justement JS, Moir S, Hallahan CW, Maenza J, et al (2007) Decay of the HIV reservoir in patients receiving antiretroviral therapy for extended periods: implications for eradication of virus. J Infect Dis 195: 1762–1764. 17492591 10.1086/518250

[15] Koopman JS, Jacquez JA, Welch GW, Simon CP, Foxman B, et al (1997) The role of early HIV infection in the spread of HIV through populations. J Acquir Immune Defic Syndr Hum Retrovirol 14: 249–258. 9117458 10.1097/00042560-199703010-00009

[16] Moir S, Buckner CM, Ho J, Wang W, Chen J, et al (2010) B cells in early and chronic HIV infection: evidence for preservation of immune function associated with early initiation of antiretroviral therapy. Blood 116: 5571–5579. 20837780 10.1182/blood-2010-05-285528 PMC3031405

[17] Rosenberg ES, Altfeld M, Poon SH, Phillips MN, Wilkes BM, et al (2000) Immune control of HIV-1 after early treatment of acute infection. Nature 407: 523–526. 11029005 10.1038/35035103

[18] Lavreys L, Thompson ML, Martin HL Jr, Mandaliya K, Ndinya-Achola JO, et al (2000) Primary human immunodeficiency virus type 1 infection: clinical manifestations among women in Mombasa, Kenya. Clin Infect Dis 30: 486–490. 10722432 10.1086/313718

[19] Powers KA, Miller WC, Pilcher CD, Mapanje C, Martinson FE, et al (2007) Improved detection of acute HIV-1 infection in sub-Saharan Africa: development of a risk score algorithm. AIDS 21: 2237–2242. 18090052 10.1097/QAD.0b013e3282f08b4d PMC2673577

[20] Kahn JO, Walker BD (1998) Acute human immunodeficiency virus type 1 infection. N Engl J Med 339: 33–39. 9647878 10.1056/NEJM199807023390107

[21] Cohen MS, Gay CL, Busch MP, Hecht FM (2010) The detection of acute HIV infection. J Infect Dis 202 Suppl 2S270–277. 20846033 10.1086/655651

[22] Lindback S, Thorstensson R, Karlsson AC, von Sydow M, Flamholc L, et al (2000) Diagnosis of primary HIV-1 infection and duration of follow-up after HIV exposure. Karolinska Institute Primary HIV Infection Study Group. AIDS 14: 2333–2339. 11089621 10.1097/00002030-200010200-00014

[23] Stacey AR, Norris PJ, Qin L, Haygreen EA, Taylor E, et al (2009) Induction of a striking systemic cytokine cascade prior to peak viremia in acute human immunodeficiency virus type 1 infection, in contrast to more modest and delayed responses in acute hepatitis B and C virus infections. J Virol 83: 3719–3733. 19176632 10.1128/JVI.01844-08 PMC2663284

[24] Rosenberg NE, Kamanga G, Phiri S, Nsona D, Pettifor A, et al (2012) Detection of acute HIV infection: a field evaluation of the determine(R) HIV-1/2 Ag/Ab combo test. J Infect Dis 205: 528–534. 22207651 10.1093/infdis/jir789 PMC3318673

[25] van Loggerenberg F, Mlisana K, Williamson C, Auld SC, Morris L, et al (2008) Establishing a cohort at high risk of HIV infection in South Africa: challenges and experiences of the CAPRISA 002 acute infection study. PLoS ONE 3: e1954. 18414658 10.1371/journal.pone.0001954 PMC2278382

[26] Francois van Loggerenberg AAD, Magdalena E. Sobieszczyk, Lise Werner, Anneke Grobler, and Koleka Mlisana, for the CAPRISA 002 Acute Infection Study Team (2012) HIV prevention in high-risk women in South Africa: Condom use and the need for change. 10.1371/journal.pone.0030669 PMC3281865 22363467

[27] Kaufmann GR, Cunningham P, Zaunders J, Law M, Vizzard J, et al (1999) Impact of early HIV-1 RNA and T-lymphocyte dynamics during primary HIV-1 infection on the subsequent course of HIV-1 RNA levels and CD4+ T-lymphocyte counts in the first year of HIV-1 infection. Sydney Primary HIV Infection Study Group. J Acquir Immune Defic Syndr 22: 437–444. 10961604 10.1097/00126334-199912150-00003

[28] Kaufmann GR, Duncombe C, Zaunders J, Cunningham P, Cooper D (1998) Primary HIV-1 infection: a review of clinical manifestations, immunologic and virologic changes. AIDS Patient Care STDS 12: 759–767. 11362020 10.1089/apc.1998.12.759

[29] Lyles RH, Munoz A, Yamashita TE, Bazmi H, Detels R, et al (2000) Natural history of human immunodeficiency virus type 1 viremia after seroconversion and proximal to AIDS in a large cohort of homosexual men. Multicenter AIDS Cohort Study. J Infect Dis 181: 872–880. 10720507 10.1086/315339

[30] Niu MT, Stein DS, Schnittman SM (1993) Primary human immunodeficiency virus type 1 infection: review of pathogenesis and early treatment intervention in humans and animal retrovirus infections. J Infect Dis 168: 1490–1501. 8245534 10.1093/infdis/168.6.1490

[31] Schacker T, Collier AC, Hughes J, Shea T, Corey L (1996) Clinical and epidemiologic features of primary HIV infection. Ann Intern Med 125: 257–264. 8678387 10.7326/0003-4819-125-4-199608150-00001

[32] Tindall B, Barker S, Donovan B, Barnes T, Roberts J, et al (1988) Characterization of the acute clinical illness associated with human immunodeficiency virus infection. Arch Intern Med 148: 945–949. 3258508

[33] Pilcher CD, Price MA, Hoffman IF, Galvin S, Martinson FE, et al (2004) Frequent detection of acute primary HIV infection in men in Malawi. AIDS 18: 517–524. 15090805 10.1097/00002030-200402200-00019

[34] Serna-Bolea C, Munoz J, Almeida JM, Nhacolo A, Letang E, et al (2010) High prevalence of symptomatic acute HIV infection in an outpatient ward in southern Mozambique: identification and follow-up. AIDS 24: 603–608. 20019574 10.1097/QAD.0b013e328335cda3

[35] Ndung’u T, Sepako E, McLane MF, Chand F, Bedi K, et al (2006) HIV-1 subtype C in vitro growth and coreceptor utilization. Virology 347: 247–260. 16406460 10.1016/j.virol.2005.11.047

[36] Welz T, Hosegood V, Jaffar S, Batzing-Feigenbaum J, Herbst K, et al (2007) Continued very high prevalence of HIV infection in rural KwaZulu-Natal, South Africa: a population-based longitudinal study. AIDS 21: 1467–1472. 17589193 10.1097/QAD.0b013e3280ef6af2

[37] Henrard DR, Daar E, Farzadegan H, Clark SJ, Phillips J, et al (1995) Virologic and immunologic characterization of symptomatic and asymptomatic primary HIV-1 infection. J Acquir Immune Defic Syndr Hum Retrovirol 9: 305–310. 7788430

[38] Mellors JW, Rinaldo CR Jr, Gupta P, White RM, Todd JA, et al (1996) Prognosis in HIV-1 infection predicted by the quantity of virus in plasma. Science 272: 1167–1170. 8638160 10.1126/science.272.5265.1167

[39] Vanhems P, Hirschel B, Phillips AN, Cooper DA, Vizzard J, et al (2000) Incubation time of acute human immunodeficiency virus (HIV) infection and duration of acute HIV infection are independent prognostic factors of progression to AIDS. J Infect Dis 182: 334–337. 10882619 10.1086/315687

[40] Ghosn J, Deveau C, Chaix ML, Goujard C, Galimand J, et al (2010) Despite being highly diverse, immunovirological status strongly correlates with clinical symptoms during primary HIV-1 infection: a cross-sectional study based on 674 patients enrolled in the ANRS CO 06 PRIMO cohort. J Antimicrob Chemother 65: 741–748. 20167586 10.1093/jac/dkq035

[41] Lavreys L, Baeten JM, Chohan V, McClelland RS, Hassan WM, et al (2006) Higher set point plasma viral load and more-severe acute HIV type 1 (HIV-1) illness predict mortality among high-risk HIV-1-infected African women. Clin Infect Dis 42: 1333–1339. 16586394 10.1086/503258

[42] Lavreys L, Baeten JM, Overbaugh J, Panteleeff DD, Chohan BH, et al (2002) Virus load during primary Human Immunodeficiency Virus (HIV) type 1 infection is related to the severity of acute HIV illness in Kenyan women. Clin Infect Dis 35: 77–81. 12060878 10.1086/340862

[43] Cohen T, Corbett EL (2011) Test and treat in HIV: success could depend on rapid detection. Lancet 378: 204–206. 21684592 10.1016/S0140-6736(11)60896-9

